# Genome shuffling enhances stress tolerance of *Zymomonas mobilis* to two inhibitors

**DOI:** 10.1186/s13068-019-1631-4

**Published:** 2019-12-16

**Authors:** Weiting Wang, Bo Wu, Han Qin, Panting Liu, Yao Qin, Guowei Duan, Guoquan Hu, Mingxiong He

**Affiliations:** 1 Biomass Energy Technology Research Centre, Key Laboratory of Development and Application of Rural Renewable Energy (Ministry of Agriculture and Rural Affairs), Biogas Institute of Ministry of Agriculture and Rural Affairs, Section 4-13, Renmin Rd. South, Chengdu, 610041 People’s Republic of China; 2 Graduate School of Chinese Academy of Agricultural Science, Beijing, 100081 People’s Republic of China; 3 College of Pharmacy and Biological Engineering, Chengdu University, Chengdu, 610041 People’s Republic of China

**Keywords:** Electrofusion, Genome shuffling, Mutagenesis, Inhibitor tolerance, *Zymomonas mobilis*

## Abstract

**Background:**

Furfural and acetic acid are the two major inhibitors generated during lignocellulose pretreatment and hydrolysis, would severely inhibit the cell growth, metabolism, and ethanol fermentation efficiency of *Zymomonas mobilis*. Effective genome shuffling mediated by protoplast electrofusion was developed and then applied to *Z. mobilis*.

**Results:**

After two rounds of genome shuffling, 10 different mutants with improved cell growth and ethanol yield in the presence of 5.0 g/L acetic acid and 3.0 g/L furfural were obtained. The two most prominent genome-shuffled strains, 532 and 533, were further investigated along with parental strains in the presence of 7.0 g/L acetic acid and 3.0 g/L furfural. The results showed that mutants 532 and 533 were superior to the parental strain AQ8-1 in the presence of 7.0 g/L acetic acid, with a shorter fermentation time (30 h) and higher productivity than AQ8-1. Mutant 533 exhibited subtle differences from parental strain F34 in the presence of 3.0 g/L furfural. Mutations present in 10 genome-shuffled strains were identified via whole-genome resequencing, and the source of each mutation was identified as either de novo mutation or recombination of the parent genes.

**Conclusions:**

These results indicate that genome shuffling is an efficient method for enhancing stress tolerance in *Z. mobilis*. The engineered strains generated in this study could be potential cellulosic ethanol producers in the future.

## Background

Lignocellulosic biomass, the most abundant, inexpensive alternative to food crop resources, represents a possible feedstock for renewable chemicals and fuels [1]. However, inhibitors (furans, weak acids and phenols) are inevitably formed during pretreatment and hydrolysis of lignocellulosic substrates [2] and are harmful to *Zymomonas mobilis* growth and ethanol fermentation. Furfural and acetic acid are the two major inhibitors present in lignocellulosic hydrolysates. Furfural is considered the most potent inhibitor owing to its high abundance (1.0–5.0 g/L), strong toxicity and synergistic effect with other inhibitors. Growth of wild *Z. mobilis* ZM4 was significantly inhibited by 1.5 g/L furfural, and furfural affected *Z. mobilis* cells in many ways, including destruction of membrane integrity, conversion of dsDNA to ssDNA as a mutagen, a decrease in NADH and ATP concentrations, and direct restriction of intracellular central carbon metabolism [3–5]. Acetic acid is the most abundant weak acid in lignocellulosic hydrolysates, and its concentration can reach 10.0 g/L [6]. However, the growth of wild *Z. mobilis* ZM4 was significantly inhibited by 3.0 g/L acetic acid. Acetic acid affects *Z. mobilis* cell growth in several ways, including decreasing the pH (disrupting the function of cellular membranes), inducing the accumulation of reactive oxygen species (ROS), increasing the lag phase duration and diverting ATP from cellular growth and maintenance [7, 8]. One way to overcome furfural and acetic acid inhibitors is to physically or chemically remove them from the biomass after pretreatment, which requires additional equipment and increases the overall production cost [9]. Another method utilizes inhibitor-tolerant strains, which is a cost-effective method.

Currently, the known metabolic furfural detoxification mechanism involves converting furfural to the less toxic compounds furyl alcohol and furyl acid through NAD(P)H-dependent reductive pathways [10–12]. Due to limited knowledge of furfural toxicity towards *Z. mobilis*, most reviews have focused on random mutation method, and few have addressed by genetic manipulation. For instance, through three rounds of adaptive laboratory evolution (ALE), the mutant ZMF3-3 was selected and demonstrated to tolerate 3.0 g/L furfural [15]. Error-prone PCR of the *Rpo*D gene (global transcription factor) in *Z. mobilis* ZM4 resulted in a mutant, ZM4-MF2, which tolerated 3.0 g/L furfural [13]. Mutants F211 and F27, resulting from error-prone PCR-based whole-genome shuffling of *Z. mobilis* CP4, also survived in 3.0 g/L furfural [14].

Besides, the overexpression of ZMO0976 (putative aldose reductase) and ZMO1771 (NADPH-dependent alcohol dehydrogenase) in *Z. mobilis* have been reported to be responsible for converting furfural to the less toxic compound furyl alcohol [16, 17].

Acetic acid enters cells via passive diffusion and dissociates into an acetate anion and a proton, lowering the pH and causing an accumulation of anions, *Z. mobilis* cells expel protons via plasma membrane H^+^-ATPase, which is driven by abundant ATP used in cellular growth and metabolic cycles [7]. Rational modifications and random mutagenesis have been applied to enhance acetic acid tolerance in *Z. mobilis.* Expression of the exogenous gene *Pbp*, a 24-amino acid proton-buffering peptide, could improve the transient tolerance of *Z. mobilis* CP4 to low pH and acids [18]. Via multiplex atmospheric and room-temperature plasma (mARTP) mutagenesis, the two *Z. mobilis* mutants AQ8-1 and AC8-9, which could tolerate 8 g/L acetic acid, and the mutant PH1-29, which could tolerate pH 3.5, were generated [19].

Furthermore, some attempts to treat multi-inhibitor resistance have been successful. The transcriptional regulator *hfq*, an RNA-binding protein, has been confirmed to enhance cellular tolerance against furfural and acetate in experiments involving its knockout mutant, AcR [20]. The flocculant mutant strain ZM401, which has improved tolerance to inhibitory compounds, especially acetic acid and vanillin, in the hydrolysate, was selected through *N*-methyl-*N*-nitro-*N*-nitrosoguanidine (NTG) mutagenesis [21]. However, although rational metabolic engineering and classical non-recombinant methods are effective for improving the resistance of phenotypes of *Z. mobilis* strains to inhibitors, lignocellulosic hydrolysates are complicated and contain furfural, weak acids, and vanillin together. The levels of tolerance reached by genetic manipulation, i.e., editing one or more genes, are not sufficient for the high concentrations of inhibitors present in lignocellulosic hydrolysates, and most genetic manipulations were performed for only one phenotype. Traditional methods for generating *Z. mobilis* strains that are resistant to inhibitors are generally laborious and time-consuming. Thus, we attempted to improve the stress resistance of *Z. mobilis* using an effective genome shuffling method, which has been successfully applied in rapid strain improvement for both eukaryotic and prokaryotic cells [22–29].

Genome shuffling is a powerful technique for rapid phenotypic improvement and recombines whole genomes of selected multi-parental strains with protoplast fusion. The classic mutagenesis approach requires a long period of continuous screening, rarely obtains strains with multiple excellent traits and after repeated rounds of mutagenesis, shows very little improvement in production. Genome shuffling, after iterative rounds of genome recombination, eliminates negative mutations and increases productivity, thus greatly making up for the defects of the classical mutagenesis method [23, 26]. Two rounds of genome shuffling in *Streptomyces fradiae* increased its production of the antibiotic tylosin; in contrast, this increase required 20 rounds of classical strain improvement (CSI) [26]. After five rounds of genome shuffling, *Lactobacillus* not only withstood acid stress (pH 4.0), but also produced threefold more lactic acid than the wild type [24]. Three different mutagens improved yields of four lipopeptides in *Bacillus amyloliquefaciens* strains that were obtained from parental strains after two rounds of genome shuffling; a high-yielding strain that produced 179.22 mg/L lipopeptides was selected [27]. Additionally, genome shuffling is widely applied in *Saccharomyces cerevisiae, Clostridium acetobutylicum* and *Lactobacillus delbrueckii* [22, 28, 29].

Recently, our group first used mARTP mutagenesis on *Z. mobilis* and obtained the most acetic acid-resistant strain, AQ8-1, which can tolerate 8.0 g/L acetic acid [19], and F34, which can resist the stress from 3.0 g/L furfural. We attempted to use genome shuffling to enhance the tolerance of AQ8-1 and F34 to two inhibitors (furfural and acetic acid), and after two rounds of genome shuffling, 10 mutants that could tolerate 5.0 g/L acetic acid and 3.0 g/L furfural were obtained.

## Results

### Tolerance characteristics of parental strains

Strain AQ8-1 grew in 5.0 g/L acetic acid with 2.0 g/L furfural and in 6.0 g/L acetic acid with 1.5 g/L furfural within 5 days and in 7.0 g/L acetic acid with 1.0 g/L furfural within 10 days. Furthermore, strain F34 grew in 5.0 g/L acetic acid with 1.0 g/L furfural and in 3.0 g/L acetic acid with 1.5 g/L furfural within 5 days and in 2.0 g/L acetic acid with 2.5 g/L furfural within 10 days. However, ZM4 only grow in 3.0 g/L acetic acid with 1.0 g/L furfural within 5 days and in 3.0 g/L acetic acid with 1.5 g/L furfural within 10 days. Although these three strains were resistant to both acetic acid and furfural, AQ8-1 showed higher tolerance to furfural than F34 and the wild-type strain ZM4 under acetic acid stress (Additional file 1: Figure S1).

### Protoplast electrofusion

The fusion of *Z. mobilis* cells occurs in two steps: cells adhere to each other, aligning themselves in a line, and then they fuse. Each process requires optimal conditions to ensure fusion success. The main function of an alternating current (AC) is to induce dielectrophoretic force and polarization of cells. In this study, when the AC field strength is set to 400 V/cm, up to 90% of the cells can be aligned in a chain, then after the DC was applied, the cells began to change form, becoming oblong in alignment with the direction of the two electrodes, the cells membrane fused and the genomes of these cells are shuffled. The last column of Additional file 1: Table S1 shows the fusion rate for each run. The range analysis in Additional file 1: Table S1 shows that the optimal electrofusion conditions were as follows: pulse-field density 8 kV/cm (A2), pulse time 25 μs (B2), and pulse number 3 times (C3). The *R*-value represented the effects of the three factors, and the effect order was as follows: pulse-field density > pulse number > pulse time.

### Screening of fusions

**Fig. 1:**
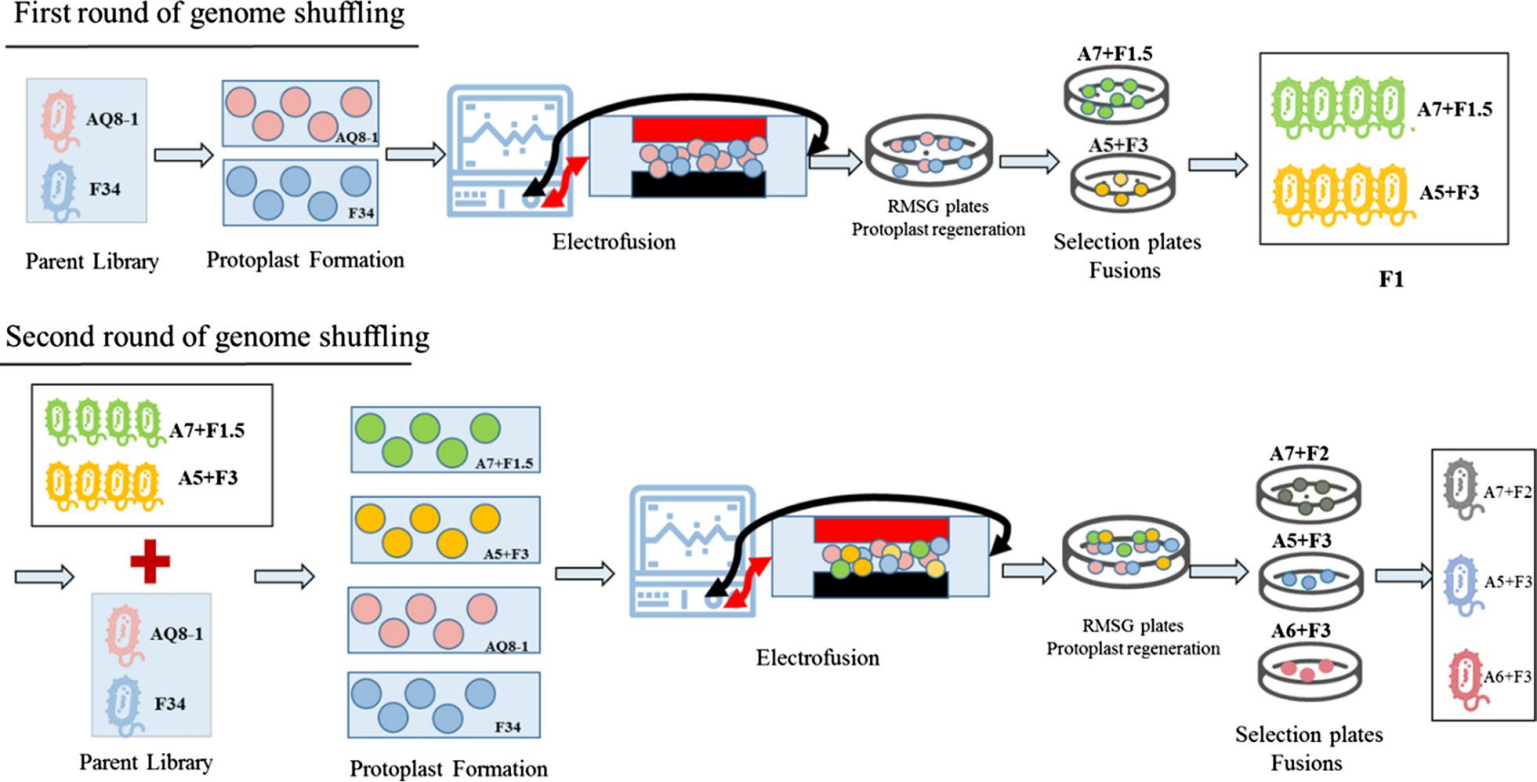
The procedure of genome shuffling for improved resistant to acetic acid and furfural of *Zymomonas mobilis*. Details on the mutagenesis were described in “Materials and methods” and “Results”

Depending on the parental strains’ resistance to the two inhibitors, we designed four gradient plates for screening: 7.0 g/L acetic acid and 1.5 g/L furfural, 6.0 g/L acetic acid and 2.0 g/L furfural, 5.0 g/L acetic acid and 3.0 g/L furfural, and 3 g/L acetic acid with 2.5 g/L furfural. After each test of electrofusion conditions, recombinants grown on regeneration plates (RMSG agar plates) were washed and diluted to OD_600_ = 1.0 (10^6^ cells/mL), and then 100.0 μL was spread on screening plates with parental strains and wild-type strain ZM4 for contrast. After 1 week, 39, 1, 4 and 563 clones survived on 7.0 g/L acetic acid and 1.5 g/L furfural, 6.0 g/L acetic acid and 2.0 g/L furfural, 5.0 g/L acetic acid and 3.0 g/L furfural, and 3.0 g/L acetic acid with 2.5 g/L furfural plates, respectively. After these clones were cultured in RM liquid medium with the corresponding concentrations of inhibitors for 10 generations, 20 fusions that always showed resistance to 7.0 g/L acetic acid and 1.5 g/L furfural and 4 fusions that always showed resistance to 5.0 g/L acetic acid and 3.0 g/L furfural were used for further screening. Based on the results of cell growth, the 4 strains that grew best with 7.0 g/L acetic acid and 1.5 g/L furfural (data not shown) were retained, and the 4 fusions withstanding 5.0 g/L acetic acid and 3.0 g/L furfural were denoted F1. Then, the F1 strains and parental strains AQ8-1 and F34 served as the starting pool for the second round of mating and selection. We chose 8.0 g/L acetic acid and 1.0 g/L furfural, 7.0 g/L acetic acid and 2.5 g/L furfural, 7.0 g/L acetic acid and 2.0 g/L furfural, 6.0 g/L acetic acid and 3.0 g/L furfural, and 5.0 g/L acetic acid with 3.0 g/L furfural as selection concentration combinations. Surprisingly, we obtained one clone tolerant to 8.0 g/L acetic acid and 1.0 g/L furfural. However, this strain grew only on a solid plate, but in liquid medium with 8.0 g/L acetic acid and 1.0 g/L furfural. At the same time, we obtained three additional clones on 7.0 g/L acetic acid and 2.5 g/L furfural, and these strains showed poor stability after 5 generations. We successfully obtained 10, 3 and 3 clones against 7.0 g/L acetic acid and 2.0 g/L furfural, 6.0 g/L acetic acid and 3.0 g/L furfural, and 5.0 g/L acetic acid with 3.0 g/L furfural, respectively. All strains showed good stability and growth performance under these stress conditions. In particular, strains against 5.0 g/L acetic acid and 3.0 g/L furfural grew well on the corresponding solid plates only after 4 days. In summary, after two rounds of genome shuffling, we obtained 20, 10, 3 and 7 strains against 7.0 g/L acetic acid and 1.5 g/L furfural, 7.0 g/L acetic acid and 2.0 g/L furfural, 6.0 g/L acetic acid and 3.0 g/L furfural, and 5.0 g/L acetic acid with 3.0 g/L furfural, respectively (Fig. 1).

Furfural is derived from the over-degradation of pentose during lignocellulose pretreatment. The high abundance of furfural can cause strong toxicity and synergistic inhibition with other inhibitors, the reported highest concentrations of furfural *Z. mobilis* could tolerate was 3.0 g/L. We chose fusions that could tolerate 3.0 g/L furfural for further analysis of fermentation and whole-genome sequencing. Ten strains were involved, including the first round of electrofusion strains against 5.0 g/L acetic acid and 3.0 g/L furfural (strains 271, 272, 273 and 274), the second round of electrofusion strains against 5.0 g/L acetic acid and 3.0 g/L furfural (strains 411, 532, and 533), and the second round of electrofusion strains against 6.0 g/L acetic acid with 3.0 g/L furfural (strains 631, 633, and 635), and the relative performances of the best-performing strains, 532 and 533, were analyzed using the inhibitors furfural and acetic acid individually and in combination.

### Profiling of cell growth and ethanol yield under different stress conditions

**Fig. 2:**
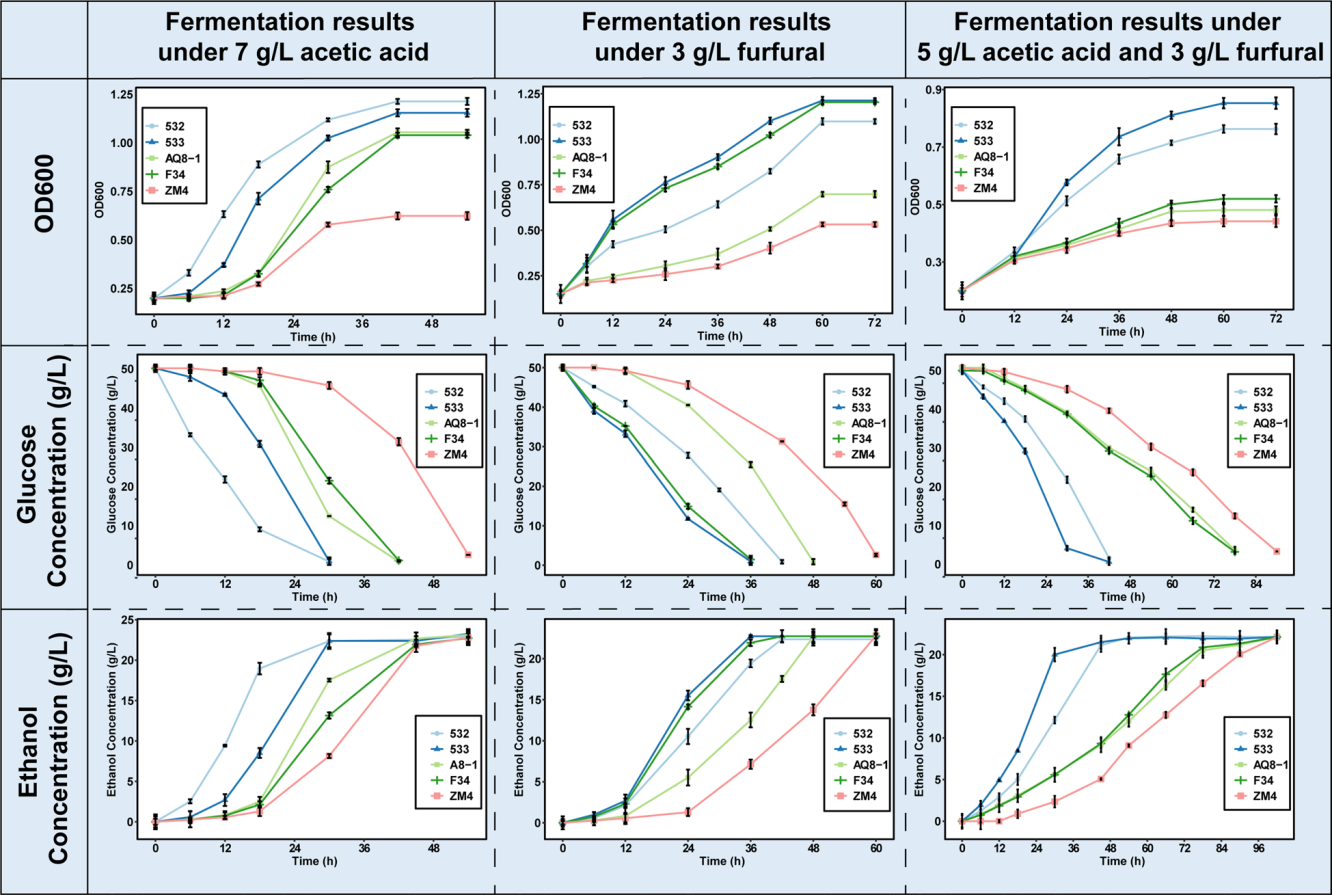
Conversion of glucose to ethanol in *Z. mobilis* under acetic acid and/or furfural stresses. Growth is indicated by OD_600_ value, glucose indicates the concentration of the sugar remained in cultures, EtOH indicates the concentration of ethanol produced. Three replicates were performed for each strain, and error bars indicate standard deviation

First, 532, 533, parental strains AQ8-1 and F34, and wild-type strain ZM4 were investigated with 5.0 g/L acetic acid and 3.0 g/L furfural. In the case of the same initial OD_600_, when strains were cultivated for up to 36 h, the OD_600_ values of 532 and 533 were increased by 76.8 ± 19.1% compared with the wild-type strain ZM4 (Fig. 2c). Besides, 532 and 533 consumed 97.8 ± 1.4% of the initial glucose within 42 h, while the parental strains AQ8-1 and F34 consumed only 40.9 ± 2.0% of the glucose and ZM4 consumed 20.8 ± 1.2% of the glucose (Fig. 2f). The ethanol productivities of 532 and 533 were 0.5 ± 0.01 and 0.51 ± 0.01 g/L/h, which were 88.5 ± 19.5% higher than that of parental strains, and 130.5 ± 17.5% higher than that of wild-type strain ZM4 (Fig. 2i and Table 1). These results indicate that 532 and 533 truly exceeded the parental strains AQ8-1 and F34 in their tolerance and showed excellent fermentation performance in the presence of the two inhibitors.

**Table 1:** Conversion of glucose to ethanol in *Z. mobilis* under acetic acid and/or furfural stresses

Strain	Fermentation time (h)	Glucose consumed (g/L)	Ethanol			Theoretical value ratio (%)
			Titer (g/L)	Yield (g/g glucose)	Productivity (g/L/h)	
50 g/L glucose + 7 g/L acetic acid						
533	30	50.23 ± 0.22	23.26 ± 1.16	0.463 ± 0.02	0.77 ± 0.04***	90.0
532	30	50.26 ± 0.17	22.96 ± 0.38	0.456 ± 0.01	0.76 ± 0.01***	89.2
AQ8-1	42	50.37 ± 0.28	23.13 ± 0.27	0.459 ± 0.00	0.55 ± 0.01***	89.8
F34	48	50.27 ± 0.05	22.80 ± 0.99	0.453 ± 0.02	0.48 ± 0.02**	88.6
ZM4	60	50.46 ± 0.18	22.73 ± 0.49	0.450 ± 0.01	0.38 ± 0.01	88.1
50 g/L glucose + 3 g/L furfural						
533	36	50.25 ± 0.39	22.73 ± 1.32	0.452 ± 0.02	0.63 ± 0.04***	88.5
532	36	50.13 ± 0.24	22.37 ± 0.54	0.446 ± 0.01	0.62 ± 0.02***	87.2
AQ8-1	48	50.24 ± 0.09	22.67 ± 0.26	0.451 ± 0.00	0.47 ± 0.01***	88.2
F34	36	50.36 ± 0.22	22.73 ± 0.17	0.451 ± 0.00	0.63 ± 0.00***	88.2
ZM4	72	50.52 ± 0.07	22.72 ± 0.17	0.449 ± 0.00	0.31 ± 0.00	87.8
50 g/L glucose + 5 g/L acetic acid + 3 g/L furfural						
533	42	50.72 ± 0.17	21.49 ± 0.59	0.423 ± 0.01*	0.51 ± 0.01***	82.8
532	42	50.81 ± 0.05	21.14 ± 0.24	0.416 ± 0.00	0.50 ± 0.01***	81.4
AQ8-1	78	50.63 ± 0.02	20.51 ± 0.16	0.405 ± 0.00	0.26 ± 0.00***	79.3
F34	78	50.54 ± 0.11	20.86 ± 1.32	0.412 ± 0.03	0.27 ± 0.02*	80.6
ZM4	90	50.72 ± 0.09	20.03 ± 0.52	0.395 ± 0.01	0.22 ± 0.01	77.2

Three repeats were performed for each strain, and error bars indicated standard deviation

*P* values calculated by one-way ANOVA, * *P* < 0.05; ** *P* < 0.01;*** *P* < 0.001

Second, we tested 532, 533, parental strains AQ8-1 and F34, and wild-type strain ZM4 in single-inhibitor condition (7.0 g/L acetic acid). Strains 532 and 533 performed better than the parental strain AQ8-1. Strains 532 and 533 were observed to grow after 6 h of culture, while AQ8-1 was observed to grow after 12 h. With the same initial OD_600_, when 532 and 533 were cultured for 18 h, their cell densities increased by 154 ± 35% compared with AQ8-1 (Fig. 2a). Strains 532 and 533 consumed 98.07 ± 0.4% of the initial glucose after fermentation for 30 h, and in less than 12 h, AQ8-1 consumed 98.2 ± 0.1% of the glucose (Fig. 2d and Table 1).

Third, we further tested 532 and 533 under 3.0 g/L furfural and compared their performances with those of the parental strains AQ8-1 and F34 and the wild-type strain ZM4. The growth performance of 533 was slightly better than that of F34, and the growth performance of 532 was higher only than that of AQ8-1 and ZM4. With the same initial OD_600_, after fermentation for 48 h, the OD_600_ of 532 and 533 were increased by 144 ± 57% compared with wild-type strain ZM4 (Fig. 2b). The ethanol productivities of 533 and F34 were the same (Fig. 2h and Table 1), and no difference was found between 533 and F34 in glucose consumption (Fig. 2e), and these strains consumed 98.03 ± 0.3% of the initial glucose in 36 h.

### Genetic changes in genome-shuffled double-resistant strains

**Fig. 3:**
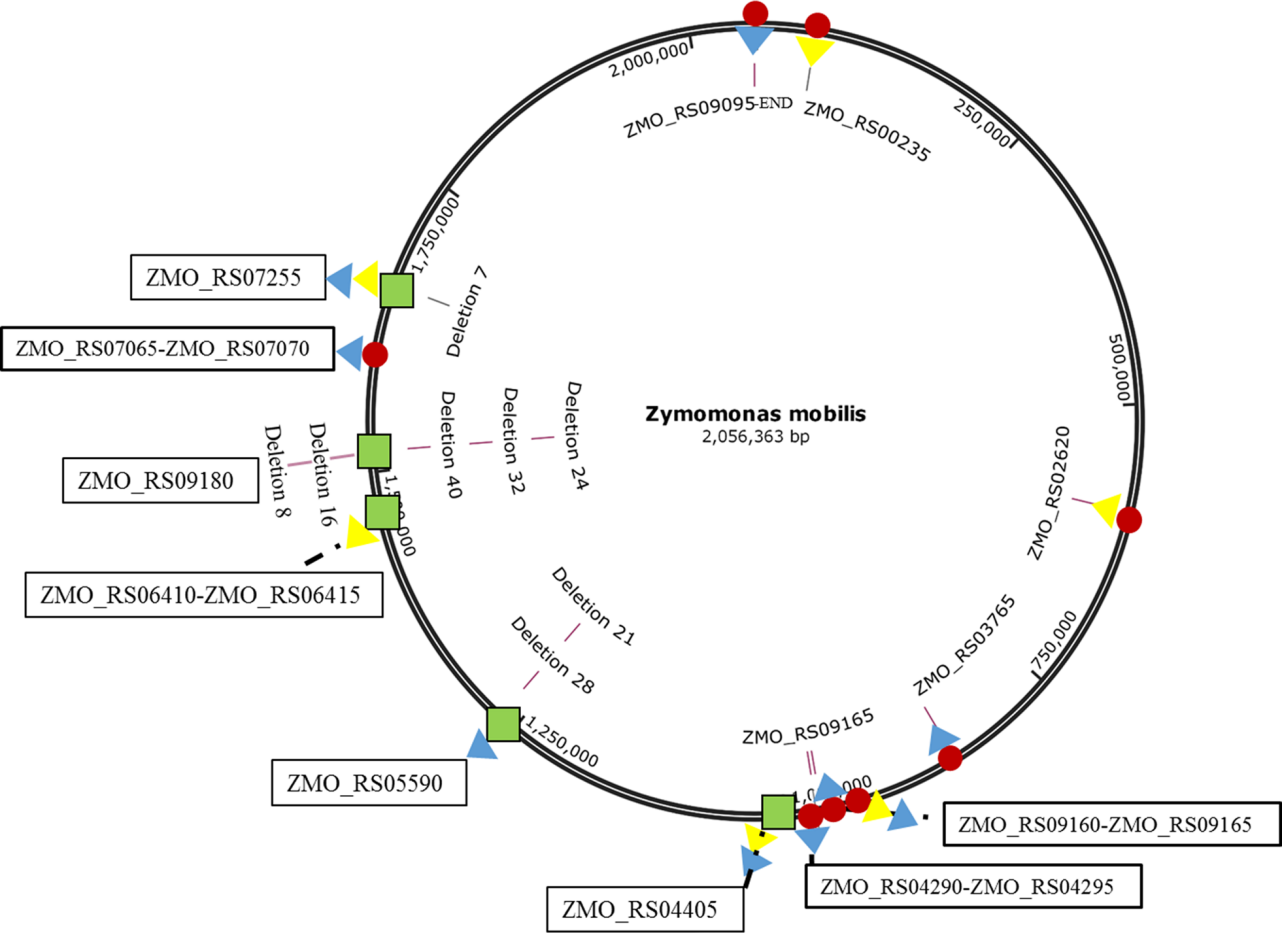
The mutation sites in 10 genome-shuffled strains compared with ZM4. The red circles represent SNVs, the green bars represent InDels, yellow triangles represent mutations derived from strain AQ8-1, and blue triangles represent mutations derived from strain F34

Multiple mutations compared to the wild-type strain ZM4 were found in each mutant strain. Nineteen identical single nucleotide variants (SNVs) were identified in all 10 mutants (Table 2), of which six were located in the coding sequence (CDS) and 13 in the intergenic regions. The SNVs in CDS regions led to synonymous and non-synonymous amino acid (AA) changes, including two non-synonymous AA changes in ZMO_RS00235 (glutamine-fructose-6-phosphate aminotransferase) and ZMO_RS02620 (DNA repair protein RadA), one non-synonymous AA change and one synonymous AA change in ZMO_RS03765 (arginine-tRNA ligase), and two non-synonymous AA changes in ZMO_RS09165 (IS5/IS1128 family transposase). Intergenic SNVs detected in all 10 mutants were targeted in regions between genes ZMO_RS09160 and ZMO_RS09165, ZMO_RS04290 and ZMO_RS04295, and ZMO_RS07065 and ZMO_RS07070. The position of the mutation sites in the genome is shown in Fig. 3.

**Table 2:** SNVs in ten genome-shuffled mutants and parental strains

Locus	Ref	SNV	Ten genome-shuffled strains	AQ8-1	F34	Gene/product
CDS						
51967	C	T	+	+	−	ZMO_RS00235/glutamine-fructose-6-phosphate aminotransferase
590452	G	A	+	+	−	ZMO_RS02620/DNA repair protein RadA
849208	C	T	+	−	+	ZMO_RS03765/arginine-tRNA ligase
849311	C	A	+	−	+	
971308	A	G	+	−	+	ZMO_RS09165/IS5/IS1182 family transposase
971369	A	G	+	−	−	
Intergenic regions						
971059	T	A	+	+	+	ZMO_RS09160-ZMO_RS09165
						IS5/IS1182 family transposase
975503	T	G	+	−	+	ZMO_RS04290-ZMO_RS04295
975506	G	A	+	−	+	Monofunctional biosynthetic peptidoglycan
975509	C	T	+	−	+	Transglycosylase/cytochrome c
975523	C	T	+	−	+	
975525	A	T	+	−	+	
975528	T	G	+	−	+	
975532	A	T	+	−	+	
975537	A	C	+	−	+	
975540	G	T	+	−	+	
975547	T	G	+	−	+	
1612575	G	A	+	−	+	ZMO_RS07065-ZMO_RS07070
						Alpha/beta hydrolase/tRNA-Met
2055763	T	C	+	−	+	ZMO_RS09095-END
						Uroporphyrinogen decarboxylase/END

+/−, the presence/absence of variation in the genome, respectively

Eleven indels were detected in 10 mutants (Table 3), of which 10 were located in the CDSs and one in the intergenic regions. Two single nucleotide deletions in the CDS of ZMO_RS04405 (ABC transporter substrate-binding protein) caused a frameshift mutation. Two distinct deletions (− 28 bp and − 21 bp) occurred in the different locations of the gene ZMO_RS05590 (hypothetical protein-coding gene) and the nucleotide sequence of the − 21 bp deletion mutant was identical to that of the 5′-end of the − 28 bp deletion mutant. Among the 11 indels detected, five independent indels (− 40 bp, − 32 bp, − 24 bp, − 16 bp, and − 8 bp) were identified in the gene ZMO_RS09180 (hypothetical protein-coding gene). It is noteworthy that the sequence of both these indels was “ACGGGCAG”, and the former 7 bp nucleotides sequence (ACGGGCA) was a mirror structure of the indel sequence. The − 7 bp deletion identified in ZMO_RS07255 (carbamoyl phosphate synthase large subunit) and the − 1 bp deletion detected in all 10 mutants occurred between ZMO_RS06410 and ZMO_RS06415. The position of the mutation sites in the genome is shown in Fig. 3.

**Table 3:** InDels in ten genome-shuffled mutants and parental strains

Locus	Type	271	272	273	274	411	532	533	631	633	635	AQ8-1	F34	Gene/product
CDS														
1002280	D1	+	−	+	−	−	−	−	+	−	−	+	+	ZMO_RS04405
1002287	D1	−	+	−	+	+	+	+	−	+	+	+	−	/ABC transporter substrate-binding protein
1266074	D28	−	−	−	+	−	+	+	+	−	−	−	−	ZMO_RS05590/hypothetical protein
1266081	D21	+	+	+	−	+	−	−	−	+	+	−	+	
1517128	D40	−	−	−	−	+	−	−	−	−	−	−	−	ZMO_RS09180/hypothetical protein
1517136	D32	−	−	−	−	−	−	+	+	−	+	−	−	
1517144	D24	−	+	−	−	−	−	−	−	−	−	−	−	
1517152	D16	−	−	+	+	−	+	−	−	+	−	−	−	
1517160	D8	+	−	−	−	−	−	−	−	−	−	−	−	
1657469	D7	+	+	+	+	+	+	+	+	+	+	+	+	ZMO_RS07255/carbamoyl phosphate synthase large subunit
Intergenic regions														
1448818	D1	+	+	+	+	+	+	+	+	+	+	+	−	ZMO_RS06410-ZMO_RS06415
														FUSC family protein/DNA polymerase III subunit delta

+/−, the presence/absence of variation in the genome, respectively

Besides, five strains (272, 273, 274, 532, and 633) experienced internal migration of chromosomes, involved the gene ZMO_RS01090, which encodes a Bap domain-containing protein (Table 4).

**Table 4:** Structural variation in different mutant strains

Strains	Post1	Post2	Type	Size
272	245,068	245,542	ITX	251
273	245,071	245,652	ITX	256
274	245,074	245,604	ITX	257
532	244,367	244,691	ITX	266
	245,058	245,690	ITX	262
633	245,065	245,594	ITX	247

*Post1* position of the front end reads anchor area, *Post2* position of the back end reads anchor area, *Size* the estimated SV size, indicates that a SV of about size is occurring between pos1–pos2, *ITX* internal migration of chromosomes

## Discussion

Tolerance to inhibitors present in hydrolysates of lignocellulosic biomass is important for the bio-production of ethanol. In this study, we successfully applied protoplast electrofusion-mediated genome shuffling to generate recombinants from the previously generated acetic acid- and furfural-tolerant *Z. mobilis* strains AQ8-1 and F34, respectively, with an aim to improve the tolerance of *Z. mobilis* to the two inhibitory by-products present in lignocellulosic hydrolysates.

Our best-performing recombinants, 532 and 533, were investigated for their relative performance in the presence of 7.0 g/L acetic acid, 3.0 g/L furfural, and 5.0 g/L acetic acid plus 3.0 g/L furfural. 532 and 533 were superior to the parental strain AQ8-1 in the presence of 7.0 g/L acetic acid, with a shorter fermentation time (30 h) and higher productivity than AQ8-1. Strains 532 and 533 utilize glucose more rapidly compared to the acetic acid-tolerant strain A7-2, which was obtained by three rounds of ALE [15], and consumed 96% of the initial glucose in the presence of 7.0 g/L acetic acid in 48 h. The flocculent mutant *Z. mobilis* ZM401 obtained by NTG mutagenesis showed strong tolerance to acetic acid. Its ethanol productivity in the presence of 8.4 g/L acetic acid reached 2.0 g/L/h [30], which is higher than those of 532 and 533 in the presence of 7.0 g/L acetic acid. In that study, the pH was adjusted to 6.0 [30], while in our study, when RM was supplemented with 7.0 g/L acetic acid, the pH was 3.92, without adjustment, for the whole fermentation. The low productivity of 532 and 533 recombinants in this study could be due to the dual effect of anion accumulation and low pH due to acetic acid supplementation. Furthermore, 532 and 533 greatly shortened the fermentation time compared with the acetic acid-tolerant strain *Z. mobilis* AC8-9 in the presence of 7.0 g/L acetic acid, which completed the fermentation of the initial 50.0 g/L glucose in 56 h [19]. Compared to the *Z. mobilis* mutants with reported resistance to high concentrations of acetic acid, mutants 532 and 533 demonstrated higher fermentation efficiency.

Recombinant 533 used glucose more quickly compared to the furfural-tolerant strain F3-3, which was obtained after three rounds of ALE and consumed 80% of the initial glucose in the presence of 3.0 g/L furfural in 48 h [15], and ZM4-MF2, which was obtained by rewiring a sigma factor (RpoD protein) [13] and consumed 92.8% glucose in the presence of 3.0 g/L furfural in 54 h.

The *Z. mobilis* mutant AcRIM0347, an acetate-tolerant strain (AcR) with an *hfq* gene insertion, is resistant to 0.75 g/L hydroxymethylfurfural (HMF), 1 g/L furfural, and 1 g/L vanillin for 16, 19, and 21 h, respectively [20]. Additionally, the mutant AcRIM0347 demonstrated resistance to inhibitors; however, the assay did not investigate the effect of mixtures of inhibitors on the inhibitor resistance of mutant AcRIM0347.

Sequencing data revealed that the 10 genome-shuffled mutants are likely to be progenies of AQ8-1 and F34. Nineteen identical SNVs were identified in all 10 mutants, and 18 of them were identical to SNVs in the parent strains AQ8-1 or F34, which demonstrated the utility of genome shuffling by combining the genetic traits of both parents. Particularly, two (2 SNVs in CDS), 15 (3 SNVs in CDS and 12 SNVs in intergenic), and one mutation were derived from AQ8-1, F34, and either AQ8-1 or F34, respectively. It is noteworthy that the intergenic SNVs detected in all 10 mutants may have been derived from strain F34. Of the 11 indels detected in 10 mutants, five could be derived from AQ8-1 or F34, and 5 independent indels in the same gene (ZMO_RS09180) cannot derive from the parental strain. In general, the SNVs or InDels mutations in 10 genome-shuffled strains, most came from the recombination between two parents.

Genome shuffling revealed that several genes likely contributed to acid/furfural tolerance. The gene ZMO_RS00235 encodes a glutamine-fructose-6-phosphate aminotransferase that was reported to be critical for cells against organic acid stress, and gene ZMO_RS02620 encodes a DNA repair protein RadA, which is essential for the survival when cells suffer from the acid stress [31]. Jeong et al. [32] found that strand breaks occurred in *E. coli O157:H7* under acid stress and that DNA integrity was maintained through physical protection by Dps and RecA-mediated repair, suggesting that DNA repair may play an important role in acid tolerance. Besides, gene ZMO_RS03765 encodes an arginine-tRNA ligase, and ZMO_RS07255 encodes a CPSase large subunit (participating in arginine biosynthesis), which are both related to arginine biosynthesis and may relate to acid stress. Ryan et al. [34] found that arginine deiminase (ADI) genes can help *Listeria monocytogenes* survive under acidic conditions and that their expression is increased at low pH and in the presence of arginine. Huang et al. [35] found that l-arginine can inhibit the biofilm formation of *Streptococcus mutants* and that although no direct evidence has indicated a role of biofilms in acid tolerance, the cell wall/membrane is essential for maintaining cellular integrity. What’s more, ZMO_RS04405 encodes an ABC transporter substrate-binding protein was reported to be critical for cells against furfural stress. Ask et al. [33] demonstrated that PDR5 and YOR1 in *S. cerevisiae* code for ABC transporters that function in the efflux of ions and xenobiotics, are under transcriptional control of Pdr1p and Pdr3p, and can probably function in transporting furfural out of the cell, thereby relieving the stress caused by this agent.

The important mutations located in the CDS regions of AQ8-1 were inherited by the 10 mutants, but mutations in the intergenic regions between ZMO_RS04270 and ZMO_RS04275 [19] were not. However, intergenic SNVs detected in all 10 mutants were targeted between ZMO_RS04290 and ZMO_RS04295 genes, which encode monofunctional biosynthetic peptidoglycan transglycosylase (MBPT) and cytochrome c, respectively. MBPT catalyzes the formation of the glycan chain in bacterial cell walls from peptidoglycan subunits; the role of MBPT is similar to the role of polymerases in DNA construction, i.e., synthesizing or repairing the cell wall and fixing mistakes in the structure of the cell wall [36], which is essential for maintaining cellular integrity and resistance to inhibitors.

To summarize, we have demonstrated that genome shuffling is an efficient method to create *Z. mobilis* mutants with enhanced tolerance to double inhibitors from the perspective of fermentation performance and that the sequences of the 10 genome-shuffled mutants contrast with those of the parental strain genomes. The phenotypes of the mutants should be a focus for future research. In the next phase of our studies, we plan to combine RNA sequencing and chromosome conformation capture methods to explore the mechanism underlying the genome-shuffled strains.

## Materials and methods

### Microorganisms and culture conditions

The *Z. mobilis* mutant strains AQ8-1 and F34 served as parental strains for genome shuffling. A concise summary of the process that led to AQ8-1 and F34 was as followed. ZM4 cells treated by the first round of ARTP mutagenesis were screened in RM supplemented with 7.0 g/L acetic acid and 3.0 g/L furfural, respectively. Two resulting mutants A7 and F34 were obtained, then A7 was selected for the second round of ARTP mutagenesis, and one resulting mutant AQ8-1 showed dramatically enhanced tolerance to 8.0 g/L acetic acid screened. The operating conditions of ARTP were input power 120 W, gas helium 10 SLM, and jet temperature 22 °C for 30 s.

The glycerol stocks of AQ8-1 and F34 were grown at 30 °C and maintained on two agar rich medium (RM) containing 50.0 g/L glucose, 10.0 g/L yeast extract, 2.0 g/L KH_2_PO_4_, 2.0 g/L MgSO_4_ and 1.0 g/L (NH_4_)_2_SO_4_. A single colony was in 5.0 mL of RM and grown overnight at 30 °C without shaking. Cell pellets were harvested by centrifuged at 3000 rpm for 4 min at 4 °C and then inoculated in 50.0 mL of RM in a 100 mL flask with or without inhibitors. All growth and fermentations were carried out in triplicate.

### Tolerance characteristics of parental strains

For further screening of genome-shuffled mutants, we first tested AQ8-1 and F34 under a gradient of acetic acid and furfural concentrations. 100.0 μL of resuscitated cultures of AQ8-1, F34, and ZM4 at the same OD_600_ (1.0) were spread on RM agar plates supplemented with different concentrations of inhibitors (Additional file 1: Figure S1) and incubated at 30 °C for 2 weeks. The tolerance of parental strains and ZM4 was tested five times independently, with the results of the plates recorded every 2 days.

### Protoplast isolation and electrofusion

Protoplasts of *Z. mobilis* were formed using a previously reported method [37]. AQ8-1 and F34 were pre-activated and then inoculated into 50.0 mL of fresh RM liquid medium in 100 mL flasks at 30 °C for 8 h without shaking. When the OD_600_ reached 1.0, cells were harvested by centrifugation at 3000×*g* for 5 min. The precipitates were washed twice with 0.01 M Tris–HCl buffer (pH 8.0) and resuspended in SMM buffer to a final concentration of 10^7^ cells/mL. Then, 0.4 mL of 3.0 mg/mL lysozyme was added per milliliter of the above cell solution, and the samples were incubated for 5 min at 37 °C. Next, 0.05 mL of 0.1 M EDTA was added, and the samples were incubated with gentle agitation for an additional 18 min, centrifuged, resuspended in 50.0 mL of RMSG (10.0 g/L glucose, 10.0 g/L yeast extract, 2.0 g/L KH_2_PO_4_, 0.05 g/L MgSO_4_, 1.0 g/L (NH_4_)_2_SO_4_, 40.0 g/L glycine and 91.09 g/L sorbitol) and incubated at 30 °C for 4 h.

Protoplasts of AQ8-1 and F34 (500.0 μL each) were mixed and centrifuged at 3000×*g* for 5 min. Precipitates were washed twice using SMM buffer and then resuspended in freshly prepared electrode buffer containing 0.5 M sorbitol and 0.2 mM CaCl_2_. The suspension (20.0 μL) was placed between separate parallel electrodes, and electrofusion was performed using a CFB16-HB Cell electrofusion device (BEX Co., Ltd., Japan). The AC was designed as 100, 200, 300, 400, 500, and 600 V/cm, duration 120 s, and determined by the length of aligned cells. The other three parameters, pulse-field density, pulse time and pulse number, were chosen as the variables tested in single-factor experiment and orthogonal design experiment with protoplast fusion rate as a response, which was calculated as follows:

$$R = M/N \times 100\% ,$$

where *M* represents the number of colonies counted on selection plates (washed from RMSG plates with saline) after electrofusion, and *N* represents the number of colonies counted on RMSG plates after electrofusion.

In the single-factor experiments, the pulse-field density was set at 6000, 8000 and 10,000 V/cm successively; pulse time was 5, 25 and 40 µs separately, pulse number was 1, 2 and 3. By comparing the fusion rate under different conditions, the best value for each factor was selected. Based on the results of the single-factor experiments, an L9 (3^3^) orthogonal test was designed to study the optimum conditions for protoplast electrofusion. The three factors were designated A, B and C and prescribed to have three levels, which were coded 1, 2 and 3 for low, intermediate and high values, respectively (Additional file 1: Table S1).

### Selection of putative genome-shuffled strains with improved inhibitor tolerance

Recombinants were washed from regeneration plates with saline and then diluted to 10^6^ cells/mL; 100.0 μL of this mixture was spread on screening RM plates containing a certain concentration of acetic acid and furfural (exceeding parental resistance), with parental strains and wild-type strain ZM4 for contrast. After incubation at 30 °C for 5 days, colonies growing on RM plates with inhibitors were assessed individually for growth in liquid RM medium (containing the corresponding concentration of acetic acid and furfural) and fermentation in 5.0% (w/v) glucose. To ensure the stability of these tentative hybrids, they were passaged for 10 generations, and the residual sugar content, fermentation rate, biomass, and ethanol production were evaluated every generation. Unstable strains were eliminated gradually, while the strains showing similar characteristics for all 10 generations were considered genetically stable and retained for further screening. Then, we selected the strains most resistant to acetic acid and furfural, labeled them F1, and along with parental strains AQ8-1 and F34, used them for the second round of electrofusion. Screening concentration was equal to or higher than that in first selection (Fig. 1).

### Analytical methods

Cells were harvested when the OD_600_ was 1.0, and then a Bacterial DNA Kit (Omega Biotek, USA) was used to isolate genomic DNA. The quality of genomic DNA was checked via 0.7% agarose gel electrophoresis run for 45 min at 120.0 V/cm.

Genomic DNA of the 10 genome-shuffled mutants and parental strain F34 was sequenced by an Illumina HiSeq instrument (Illumina, San Diego, CA, USA), and the reference genome of strain ZM4 (GenBank No. NC_006526.2) was mapped. Annotation for potential SNVs, indels, and SVs was performed by ANNOVAR (V21 Feb 2013). The sequencing was completed by GenWize, Inc. (Suzhou, China).

Fermentation supernatant was centrifuged at 13,500 rpm for 5 min, and the precipitate was discarded; the supernatant was then passed through a 0.22 μm membrane and used to determine the concentrations of glucose and ethanol in the fermentation. High-performance liquid chromatography (HPLC, Agilent 1200) was applied to assess glucose and ethanol concentrations with 5 mM H_2_SO_4_ at a flow rate of 0.6 mL/min and a column temperature of 35 °C. The injection volume was set at 20.0 μL. The cell density was determined by a spectrophotometer detector (Jingke UV765, Shanghai) at wavelength 600 nm. Differences between the fermentation profiles of each genome-shuffled strains and the control strain were tested by one-way ANOVA.

## Conclusion

Ten mutant strains that can tolerate 5.0 g/L acetic acid and 3.0 g/L furfural have been generated via two rounds of genome shuffling. In this study, we demonstrated that the protoplast electrofusion method efficiently enhances the tolerance of *Z. mobilis*. Genome re-sequencing further revealed that 10 mutants combine the genetic trait of both parents. The mutant strains generated in this study will not only serve as potential bio-ethanol producers but also help in understanding stress response and regulation in bacteria.

## Supplementary information

**Additional file 1.**

## Data Availability

The *Z. mobilis* 532 and 533 have been deposited at Guangdong Microbial Culture Center (GDMCC) under the Accession Number GDMCC60527 and 60526, respectively.

## Abbreviations

ARTP: atmospheric and room-temperature plasma
ALE: adaptive laboratory evolution
NTG: *N*-methyl-*N*-nitro-*N*-nitrosoguanidine
RM: rich medium
OD: optical density
CDS: coding sequence
SNV: single-nucleotide variation
PCR: polymerase chain reaction
HPLC: high performance liquid chromatography
RT: room temperature
SV: structural variation
ATP: adenosine triphosphate
NADH: nicotinamide adenine dinucleotide (reduced)
HMF: hydroxymethylfurfural

## References

[1] da Silva ARG, Errico M, Rong BG (2018). Systematic procedure and framework for synthesis and evaluation of bioethanol production processes from lignocellulosic biomass. Bioresour Technol Rep.

[2] Jeon YJ, Xun Z, Rogers PL (2010). Comparative evaluations of cellulosic raw materials for second generation bioethanol production. Lett Appl Microbiol.

[3] Khan QA, Hadi SM (1993). Effect of furfural on plasmid DNA. Biochem Mol Biol Int.

[4] Almeida JR, Bertilsson M, Gorwa-Grauslund MF, Gorsich S, Lidén G (2009). Metabolic effects of furaldehydes and impacts on biotechnological processes. Appl Microbiol Biotechnol.

[5] He MX, Wu B, Shui ZX, Hu QC, Wang WG, Tan FR, Tang XY, Zhu QL, Pan K, Li Q (2012). Transcriptome profiling of *Zymomonas mobilis* under furfural stress. Appl Microbiol Biotechnol.

[6] Mills TY, Sandoval NR, Gill RT (2009). Cellulosic hydrolysate toxicity and tolerance mechanisms in *Escherichia coli*. Biotechnol Biofuels.

[7] Lawford HG, Rousseau JD (2003). Cellulosic fuel ethanol: alternative fermentation process designs with wild-type and recombinant *Zymomonas mobilis*. Appl Biochem Biotechnol.

[8] Eva P (2000). Fermentation of lignocellulosic hydrolysates. I: inhibition and detoxification [Review]. Bioresour Technol.

[9] Solange IM, Inês CR (2004). Alternatives for detoxification of diluted-acid lignocellulosic hydrolyzates for use in fermentative processes: a review. Bioresour Technol.

[10] Almeida JRM, Röder A, Modig T, Laadan B, Lidén G, Gorwa-Grauslund MF (2008). NADH- vs NADPH-coupled reduction of 5-hydroxymethyl furfural (HMF) and its implications on product distribution in *Saccharomyces cerevisiae*. Appl Microbiol Biotechnol.

[11] Gutiérrez T, Ingram LO, Preston JF (2006). Purification and characterization of a furfural reductase (FFR) from *Escherichia coli* strain LYO1—an enzyme important in the detoxification of furfural during ethanol production. J Biotechnol.

[12] Koopman F, Wierckx N, de Winde JH, Ruijssenaars HJ (2010). Identification and characterization of the furfural and 5-(hydroxymethyl)furfural degradation pathways of *Cupriavidus basilensis* HMF14. Proc Natl Acad Sci USA.

[13] Tan FR, Dai LC, Wu B, Qin H, Shui ZX, Wang JL, Zhu QL, Hu QC, Ruan ZY, He MX (2015). Improving furfural tolerance of *Zymomonas mobilis* by rewiring a sigma factor RpoD protein. Appl Microbiol Biotechnol.

[14] Huang S, Xue T, Wang Z, Ma Y, He X, Hong J, Zou S, Song H, Zhang M (2018). Furfural-tolerant *Zymomonas mobilis* derived from error-prone PCR-based whole genome shuffling and their tolerant mechanism. Appl Microbiol Biotechnol.

[15] Shui ZX, Qin H, Wu B, Ruan ZY, Wang LS, Tan FR, Wang JL, Tang XY, Dai LC, Hu GQ (2015). Adaptive laboratory evolution of ethanologenic *Zymomonas mobilis* strain tolerant to furfural and acetic acid inhibitors. Appl Microbiol Biotechnol.

[16] Agrawal M, Chen RR (2011). Discovery and characterization of a xylose reductase from *Zymomonas mobilis* ZM4. Biotechnol Lett.

[17] Wang X, Gao Q, Bao J (2017). Enhancement of furan aldehydes conversion in *Zymomonas mobilis* by elevating dehydrogenase activity and cofactor regeneration. Biotechnol Biofuels.

[18] Baumler DJ, Hung KF, Bose JL, Vykhodets BM, Cheng CM, Jeong KC, Kaspar CW (2006). Enhancement of acid tolerance in *Zymomonas mobilis* by a proton-buffering peptide. Appl Biochem Biotechnol.

[19] Wu B, Qin H, Yang Y, Duan G, Yang S, Xin F, Zhao C, Shao H, Wang Y, Zhu Q (2019). Engineered *Zymomonas mobilis* tolerant to acetic acid and low pH via multiplex atmospheric and room temperature plasma mutagenesis. Biotechnol Biofuels.

[20] Yang S, Pelletier DA, Lu TY, Brown SD (2010). The *Zymomonas mobilis* regulator hfq contributes to tolerance against multiple lignocellulosic pretreatment inhibitors. BMC Microbiol.

[21] Lee JH, Skotnicki ML, Rogers PL (1982). Kinetic studies on a flocculent strain of *Zymomonas mobilis*. Biotechnol Lett.

[22] Zhang W, Geng A (2012). Improved ethanol production by a xylose-fermenting recombinant yeast strain constructed through a modified genome shuffling method. Biotechnol Biofuels.

[23] Snoek T, Picca NM, Van den BS, Mertens S, Saels V, Verplaetse A, Steensels J, Verstrepen KJ (2015). Large-scale robot-assisted genome shuffling yields industrial *Saccharomyces cerevisiae* yeasts with increased ethanol tolerance. Biotechnol Biofuels.

[24] Patnaik R, Louie S, Gavrilovic V, Perry K, Stemmer WP, Ryan CM, del Cardayre S (2002). Genome shuffling of *Lactobacillus* for improved acid tolerance. Nat Biotechnol.

[25] Wang Y, Zhang G, Zhao X, Ling J (2017). Genome shuffling improved the nucleosides production in *Cordyceps kyushuensis*. J Biotechnol.

[26] Zhang YX, Perry K, Vinci VA, Powell K, Stemmer WP, del Cardayré SB (2002). Genome shuffling leads to rapid phenotypic improvement in bacteria. Nature.

[27] Shi J, Zhu X, Lu Y, Zhao H, Lu F, Lu Z (2018). Improving iturin a production of *Bacillus amyloliquefaciens* by genome shuffling and its inhibition against *Saccharomyces cerevisiae* in orange juice. Front Microbiol..

[28] Li SB, Qian Y, Liang ZW, Guo Y, Zhao MM, Pang ZW (2016). Enhanced butanol production from cassava with *Clostridium acetobutylicum* by genome shuffling. World J Microbiol Biotechnol.

[29] John RP, Gangadharan D, Madhavan NK (2008). Genome shuffling of *Lactobacillus delbrueckii* mutant and *Bacillus amyloliquefaciens* through protoplasmic fusion for l-lactic acid production from starchy wastes. Bioresour Technol.

[30] Zhao N, Bai Y, Liu CG, Zhao XQ, Xu JF, Bai FW (2014). Flocculating *Zymomonas mobilis* is a promising host to be engineered for fuel ethanol production from lignocellulosic biomass. Biotechnol J.

[31] Zhou Q, Zhang XJ, Xu H, Xu BJ, Hua YJ (2006). RadA: a protein involved in DNA damage repair processes of *Deinococcus radiodurans* R1. Chin Sci Bull..

[32] Jeong KC, Huang KF, Baumler DJ, Byrd JJ, Kaspar CW (2008). Acid stress damage of DNA is prevented by Dps binding in *Escherichia coli* O157:H7. BMC Microbiol.

[33] Ask M, Bettiga M, Mapelli V, Olsson L (2013). The influence of HMF and furfural on redox-balance and energy-state of xylose-utilizing *Saccharomyces cerevisiae*. Biotechnol Biofuels.

[34] Ryan S, Begley M, Gahan CG, Hill C (2009). Molecular characterization of the arginine deiminase system in *Listeria monocytogenes*: regulation and role in acid tolerance. Environ Microbiol.

[35] Huang X, Zhang K, Deng M, Exterkate RAM, Liu C, Zhou X, Cheng L, Ten CJM (2017). Effect of arginine on the growth and biofilm formation of oral bacteria. Arch Oral Biol.

[36] Baker AT, Takahashi N, Chandra SB (2010). A comparative analysis of monofunctional biosynthetic peptidoglycan transglycosylase (MBPT) from pathogenic and non-pathogenic bacteria. Korea Genome Organization..

[37] Lee KJ, Seong CN (1984). Strain development of *Zymomonas mobilis* for ethanol production—optimal conditions for the spheroplast formation and regeneration. Korean Soc Appl Microbiol Biotechnol.

