# Supplementary material for: Genome shuffling enhances stress tolerance of Zymomonas mobilis to two inhibitors
Source: Biotechnol Biofuels. 2019 Dec 16;12:288. doi: 10.1186/s13068-019-1631-4 (PMC6913010; doi:10.1186/s13068-019-1631-4)
Supplement: Supplementary file 1 — Additional file 1. [file 13068_2019_1631_MOESM1_ESM.docx]

Additional file

**Genome Shuffling Enhances Stress Tolerance of**

***Zymomonas mobilis* to Two-Inhibitors**

Weiting Wang^1, 2†^, Bo Wu^1†^, Han Qin^1^, Panting Liu^1, 2^, Yao Qin^1, 3^ ,

Guowei Duan^1, 2^, Guoquan Hu^1, 2^ and Mingxiong He^1, 2^*

^1^ Biomass Energy Technology Research Centre, Key Laboratory of Development and Application of Rural Renewable Energy (Ministry of Agriculture and Rural Affairs), Biogas Institute of Ministry of Agriculture and Rural Affairs, Section 4-13, Renmin Rd. South, Chengdu 610041, P. R. China.

^2^ Graduate School of Chinese Academy of Agricultural Science, Beijing 100081, P. R. China.

^3^ College of Pharmacy and Biological Engineering, Chengdu University, Chengdu 610041, P. R. China.

^†^*These authors contributed equally to this work.*

*Corresponding author (Fax: +86-28-85242281; E-mail: [hemingxiong@caas.cn](mailto:hemingxiong@caas.cn); hemxion@hotmail.com)

Additional Table S1 Result of range analysis of orthogonal experiments.

| **No.** | **Orthogonal Design** | | | **Fusion rate** |
| --- | --- | --- | --- | --- |
|  | **A(field density(V/cm))** | **B(time/us)** | **C(number)** |  |
| 1 | 1 | 1 | 1 | 0.003 |
| 2 | 2 | 1 | 2 | 0.163 |
| 3 | 3 | 1 | 3 | 0.052 |
| 4 | 1 | 2 | 2 | 0.003 |
| 5 | 2 | 2 | 3 | 0.239 |
| 6 | 3 | 2 | 1 | 0.001 |
| 7 | 1 | 3 | 3 | 0.004 |
| 8 | 2 | 3 | 1 | 0.003 |
| 9 | 3 | 3 | 2 | 0.002 |
| K1 | 0.003 | 0.0727 | 0.002 |  |
| K2 | 0.135 | 0.081 | 0.056 |  |
| K3 | 0.0183 | 0.003 | 0.098 |  |
| R | 0.132 | 0.078 | 0.096 |  |
| The best levels | A2 | B2 | C3 |  |
| Order | A>C>B |  |  |  |

**Additional Figure S1**


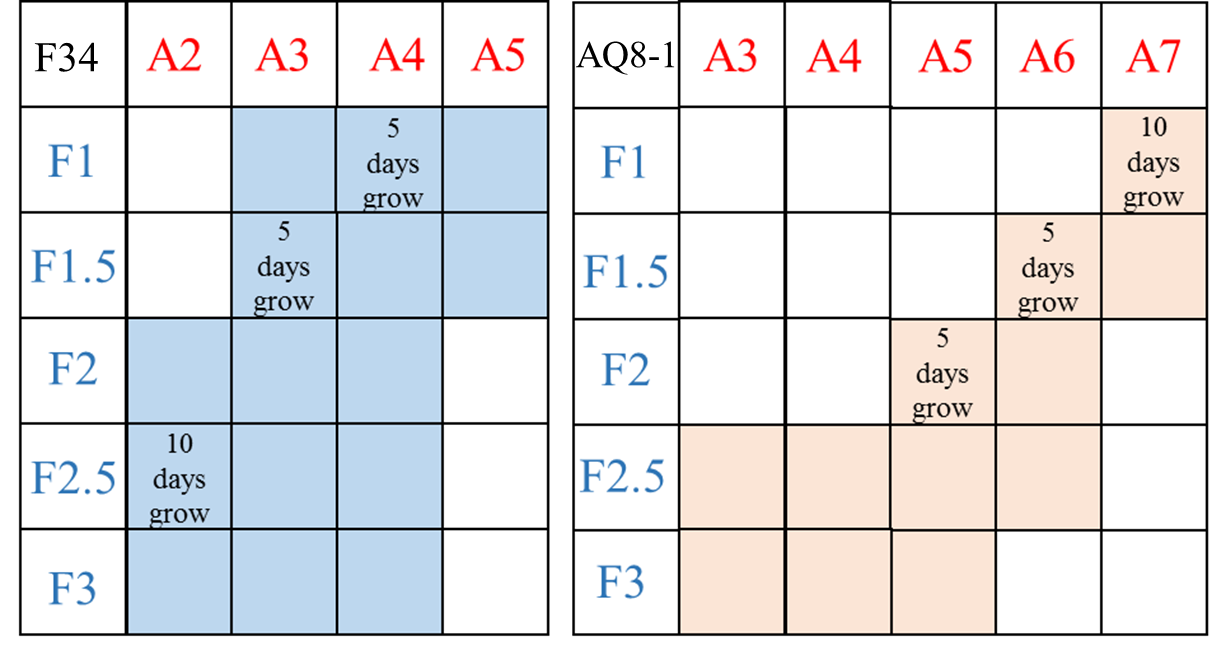


Parental strains AQ8-1 and F34 resistant to acetic acid and furfural.

Red area indicates the test combination of AQ8-1; Blue area indicates the test combination of F34.

(“An” indicates n g/L acetic acid, “Fn” indicates n g/L furfural)
